# Supplementary material for: Whole genome sequencing of Clostridioides difficile PCR ribotype 046 suggests transmission between pigs and humans
Source: PLoS One. 2020 Dec 21;15(12):e0244227. doi: 10.1371/journal.pone.0244227 (PMC7751860; doi:10.1371/journal.pone.0244227)
Supplement: Attachment — Submitted filename: Response to Reviewers.docx [file pone.0244227.s006.docx]

Response to reviewer and academic editor.

Thank you for reviewing our manuscript and for the constructive comments.

We now hope we have corrected the style requirements we missed the first time around.

Regarding the availability of data, all our data have always been deposited in the EMBL Nucleotide Sequence Database (ENA) but held private until we felt that we were close to being published and we have now made all sequence data public available. The sequences can as stated in the manuscript be found under project accession number PRJEB34857 (individual sequence accession numbers available in S1 Table).

Thank you for clarifying how you want the competing interest and funding statement to be expressed. We would like to adjust them to the following:

Funding statement:

K.J., P.M., T.N. and M.S. has received funding for this study from the Research

Committee of Region Örebro County (grant OLL-674241). The funder had no role in study design, data collection and analysis, decision to publish, or preparation of manuscript.

D.A. and F.D. employees of 1928 Diagnostics. 1928D diagnostics provided support in the form of salaries for D.A. and F.D. and made the analysis available for free, but did not have any additional role in the study design, data collection and analysis, decision to publish, or preparation of the manuscript. The specific roles of these authors are articulated in the ‘author contributions’ section.

Competing interest statement.

D.A. and F.D. are employees of 1928 Diagnostics. This does not alter our adherence to PLOS ONE policies on sharing data and materials. A.W., P.M., A.F., K.J., M.S., and T.N. declare no competing interests.

To address that PLOS ONE do not allow data not shown as reference we have added two more distances matrixes as supplementary files (S3 Table and S4 Table) covering all the data formerly referred to as data not shown.

Below we have responded to each question raised by the reviewers, for clarity the reviewer’s questions are marked in bold lettering.

**Reviewer #1:**

**This paper compares genome sequences from C. difficile ribotype 046 isolates collected from pigs and humans by using SNP typing and two different schemes for cgMLST, one of which is novel. While some of the results may be interesting, the level of methodological detail given is insufficient to fully assess the conclusions presented.**
Additional methodological information has been inserted into text as well as supplementary items for better understanding. Care has also been taken not to make the methodological issues comparing the different methods to overcast the findings of how the isolates relate to each other.

**Line 124: "For analysis in Ridom Seqsphere .... the reads were de novo assembled ... using Velvet ..." Please provide the version and parameter settings of the assembler software.**
We have changes the sentence to: “For analysis in Ridom™ SeqSphere+ the reads were *de novo* assembled through a pipeline using Velvet version 1.1.04 [17] using default settings and sequences were trimmed before assembly until the average Phred quality score was 30 in a window of 20 bases.”

**Line 127: How were the reads assembled for analysis on the 1928D platform?**
We have added the following text to clarify the assembly in the 1928D platform “The 1928 platform’s cgMLST method uses a custom developed allele calling algorithm based on an alignment free k-mer approach. For novel allele extraction, and database acceptance, local assembly is used to validate gene structure using SPAdes version 3.11.1 [18].”

**Line 137: A "cgMLST scheme based on 2,631 core genes" apparently was used for analysis with the 1928D software. This cgMLST scheme appears to be novel. However, the structure of this cgMLST scheme is not reported anywhere in the manuscript, nor is any literature reference provided. Clearly, without more detailed information on this method, the results based on this new cgMLST which are presented here are of little use to the reader. At the least, information on the genes included in the scheme and the exact positions of gene sequences in relation to a publically available reference genome sequence must be provided. It is also not clear if the novel cgMLST scheme was evaluated in any way, e.g. by applying it to a reference set of genomes or strains.**
We have added a supplementary table (S2 Table) that contains a list of all core genes in the 1928D cgMLST scheme with gene names derived from the strain 630 delta erm seed genome. And added the text: “The seed genome for picking target genes belongs to strain 630 delta erm (NCBI RefSeq assembly accession number GCF_002080065.1). The 1928 cgMLST scheme was created with the purpose to be able to analyse sequence types (STs) 1, 35, 3 and 37. ST 1 corresponds to hypervirulent RT027 [3], ST 35 corresponds to RT046 which historically has been common in Sweden [15] and STs 3 and 37 are common in certain demographic groups [20]. The scheme has also been observed to perform well for STs 81, 41, 54 and 55.” This text also contains a new reference

**Line 143: "The SNP analysis was performed using the 1928D platform" -- Unclear, which strategy was used for SNP analysis. Please provide transparent detail on the algorithm/software and parameters used.**
We have added the text “1928D’s SNP analysis pipeline uses Burrows-Wheeler Aligner version 0.7.17-r1188 [21, 22] for read alignment and FreeBayes version 1.3.2 [23] for variant calling.” and references for the software used.

**Line 146: "Variants were called at 10x minimum coverage" -- Such low coverage is usually not considered sufficient for calling SNPs. Why was a different minimum coverage set in comparison to SeqSphere cgMLST analysis (50x, see Line 129)?**
We have changed some of the wording in the sentence in line 146 to clarify, but we maintain that it is sufficient for variant calling. In the reference referred to by reviewer #1 in a comment on line 242 they used a minimum of 5 reads to call a SNP, and Bush et al. (Genomic diversity affects the accuracy of bacterial single-nucleotide polymorphism–calling pipelines, GigaScience, Volume 9, Issue 2, February 2020) also used a minimum of 5 reads, a lover threshold than ours. The minimum 50x average coverage referred to in line 129 is an average coverage for the whole genome and is used as a measurement on over all sequence quality and the minimum 10x coverage is the coverage for a specific base to ensure that we only have high quality SNP included in our analysis. To clarify this we also have changed the wording in line 129.

**Line 147: Genomic regions affected by recombination may confound SNP-based phylogenetic analyses and therefore commonly are excluded. Why did the authors choose not to do so?**
Manual analysis of recombination filtering results using Gubbins (v2.3.4) resulted in minimal variant differences that neither affected topology of the tree nor the relations between samples, the evaluation used the analysis results coming out directly from the 1928 platform.

**Line 155, Figure 1: The structure within the main clade, including all the pig isolates, cannot be discerned.**
Since the pig isolates are so closely related it is hard to visualise them in a three structure with all isolates included, it is for this reason that Fig 4 is a minimum spanning tree. We tried many different options and found that minimum spanning tree was the best way to present the data. To hopefully clarify the relationship between the isolate we also made figure 4 to include all isolates when we remade the figure in version 2 of the Ridom SeqSphere cgMLST scheme.

**Line 159, Figure 2: It is difficult to compare the trees in Figure 1 (SeqSphere) and Figure 2 (1982D), because the former was drawn in a circular format and the other was drawn rectangular. Please provide both trees in the same format.**
We have tried both configurations of the tree for figure 1 and we feel that for the Ridom SeqSphere analysis the circular is easiest to read and shows the clusters in relation to each other in a good way. Unfortunately, it is not possible to make figure 2 circular. We feel that making each figure as easy to read as possible is preferable even with the small disadvantage of having the trees configured in different ways.

**Line 233: "very similar numbers of allelic differences within the clusters" -- Are the numbers of allelic differences shown anywhere in the manuscript? This would be interesting.**
All pairwise comparisons for all three analyses are now available in S3 Table, S4 Table and S5 Table

**Line 235: "differences in the algorithms used" [by SeqSphere and 1928D] -- The algorithm used by 1928D needs to be provided.**
Please see our answers to line 127 and 143

**Line 242: "Similar performance of cgMLST and SNP analysis" -- Is this really true? Eyre et al. (J. Clin. Microbiol. 58: 01037-19) recently showed that cgMLST was inferior to SNP analysis for identifying closely related C. difficile isolates.**
The sentence in line 242 refers to other species than C. difficile but we have read the article referred to and found it a good reference to include in our article in the discussion section. It highlights some of the difficulties in analysing whole genome sequencing data and we think it strengthens our decision to include more than one analysis scheme in the article including a SNP analysis.

**Line 257: "two human isolates were intermingled in the pig and environmental cluster" -- Where and when had these human specific isolates been collected? Had they been part of the national survey, with no connection to the area around the pig farms? Please provide this information in the manuscript.**
The isolates are discussed in the results section to make the connection clearer for the reader we have named the isolates in the discussion section. The time of isolation was already named in the results section but we have also added the following text “these isolates were collected as a part of the yearly national survey and not in the same county as the pig farms are situated”.

**The sequence data is not available at ENA under the accession number indicated.**
This has been corrected as described above as a response to the editor’s questions.

**Reviewer #2:**

**Add more detailed description of cgMLST on 1928D platform. How were the sequences assembled, which assembly software was used.**
Please see our answer to reviewer #1 Line 137.

**There in an updated Ridom SeqSphere cgMLST scheme that was released recently, please re-run the analysis with the updated scheme.**
We have now reanalysed the Ridom Seqsphere analysis with version 2 of the cgMLST scheme and updated all number of allelic differences in the manuscript and updated figures 1 and 4.

**Lines 126 and 127: when were sequences trimmed, before or after assembly?**
The sequences were trimmed before assembly, this information has been added to the manuscript.

**Add more info on what parameters were used for assessing the SNV. How were the genomes assembled, were there any quality trimming applied before?**
Please see our answer to reviewer #1 Line 143

**Figure 1 and 2. Add number of different alleles between genomes. Also, mark the CC (at least for SeqSphere I know that ST that differ in less than 6 alleles can be shaded).**
To configure the figures so they are easy to read in the article is challenging and we have tried multiple configurations in Ridom SeqSphere and found that adding the requested information makes them harder to interpret. All allelic differences can be found in the now added tables S3 and S4. In Fig 4 we display the number of allelic differences in the minimum spanning trees for the Ridom SeqSphere+ analysis and the CC is shaded, we believe that from this figure the most important information can be extracted.

**To maybe improve the resolution you could also include part (core) of the accessory genome for the cgMLST analysis – this can be done using SeqSphere.**
We appreciate the suggestion and have looked in to it but we believe this to be outside the scope of this article since it would make the focus even more on compering different typing methods and our primary focus is intended to be the relationship between the isolates. We have tried analysing the isolates with the part accessory genome scheme in Ridom SeqSphere and it did not change any of our findings.

**Line 53-54: change the sentence…in this paper Eyre et al. did not unequivocally show that transmission outside the healthcare system in an important way for acquisition of CD. They suggested that there is a large, genetically diverse reservoir outside the hospital setting.**
We have changed the sentence to” and has revealed that transmission from symptomatic patients in the healthcare system only accounts for a small part of CDI cases, this suggests that asymptomatic carriage or environmental sources play an important role in the transmission of C. difficile CDI [7]”

**Add info on ENA accession number to Materials/Method section**.
As we have interpreted the submission guidelines the data availability section will be typeset with the article and to repeat the accession number in the method section feels unnecessary so at the moment we have made no changes. But if it is required to do so we will gladly add a paragraph with the accession number.

**Line 174 and 204: Change …by 0 and 204… to …from 0 to 204….**
We have changed this according to the reviewer’s suggestion.

We would like to thank the reviewers and editor for their input, we feel they have greatly improved our manuscript.

Yours sincerely

Anders Werner
